# Primary Zonal High Intensity Focused Ultrasound for Prostate Cancer: Results of a Prospective Phase IIa Feasibility Study

**DOI:** 10.1155/2014/756189

**Published:** 2014-01-23

**Authors:** Roland Van Velthoven, Fouad Aoun, Ksenija Limani, Krishna Narahari, Marc Lemort, Alexandre Peltier

**Affiliations:** ^1^Department of Urology, Jules Bordet Institute, 1 Héger-Bordet Street, 1000 Brussels, Belgium; ^2^Université Libre de Bruxelles, 50 Franklin Roosevelt Avenue, 1050 Brussels, Belgium; ^3^Department of Radiology, Jules Bordet Institute, 1 Héger-Bordet Street, 1000 Brussels, Belgium

## Abstract

*Aims.* In this study we report our results with storage of cryopreserved semen intended for preservation and subsequent infertility treatment in men with testicular cancer during the last 18 years. *Methods.* Cryopreserved semen of 523 men with testicular cancer was collected between October 1995 and the end of December 2012. Semen of 34 men (6.5%) was used for fertilization of their partners. They underwent 57 treatment cycles with cryopreserved, fresh, and/or donor sperm. *Results.* A total of 557 men have decided to freeze their semen before cancer treatment. Seminoma was diagnosed in 283 men (54.1%) and nonseminomatous germ cell tumors in 240 men (45.9%). 34 patients who returned for infertility treatment underwent 46 treatment cycles with cryopreserved sperm. Totally 16 pregnancies were achieved, that is, 34.8% pregnancy rate. *Conclusion.* The testicular cancer survivors have a good chance of fathering a child by using sperm cryopreserved prior to the oncology treatment, even when it contains only limited number of spermatozoa.

## 1. Introduction

During the last decade, proactive screening for prostate cancer (PCa) in the United States (US) and opportunistic detection in Europe led to a dramatic increase in the incidence of PCa reaching nearly 200.000 new cases per year in the US [1]. Conventional treatment options for organ confined PCa range from active surveillance to whole-gland radical therapy. Active surveillance has the distinct advantage of avoiding overtreatment and treatment related morbidity but carries the risk of silent progression of PCa in up to 35% of cases [2]. It may also induce no treatment related significant anxiety and uncertainty [3]. Radical therapy has the advantage of improving the overall and cancer specific survival in appropriately selected patients [4, 5] but bears significant risk of treatment related functional complications that detrimentally affect the quality of life [5, 6]. Therefore, counseling patients for appropriate, individual treatment strategy remains challenging even for experienced physicians.

Consequently, focal therapy has emerged as an alternative option to standard therapies. The goal of this tissue-preserving strategy as defined by the International Task Force on Prostate Cancer and the Focal Lesion Paradigm would be to “selectively ablate(s) known disease and preserve(s) existing functions, with the overall objective of minimizing lifetime morbidity without compromising life expectancy” [7]. A number of focal therapy energies and modalities have commonly been used [8]. Among these therapies, High Intensity Focused Ultrasound (HIFU) emerged as a valid mini-invasive therapy for localised prostate cancer, using focused ultrasound to generate areas of intense heat to induce tissue necrosis. This energy delivery system originally used to treat the whole prostate is used nowadays to treat a part of the gland. The ability of HIFU to achieve thermoablation of targeted prostatic lesion was proven histologically on operative specimen [9], on MRI imaging [10], and on posttreatment biopsies [11, 12].

In this paper, we aimed to target on the basis of a combined localisation strategy with multiparametric MRI and transrectal ultrasound (TRUS) guided systematic biopsy unilateral localised PCa with hemiablation HIFU. The goal of our study was to assess feasibility, safety, and short to medium term oncological and functional outcomes.

## 2. Patients and Methods

After obtaining institutional review board approval, a cohort of 31 consecutive patients with unilateral organ confined PCa primarily treated by hemiablation HIFU (from February 2007 to June 2011) were recruited into a single centre prospective phase IIa feasibility study. All patients underwent TRUS guided systematic biopsy according to the modified Gore protocol by a single experienced surgeon [13]. Patients with histologically proven unilateral PCa of any burden, PSA < 15 ng/mL, any Gleason score, no extraprostatic extension, and a clinical stage T1c-T2bN0M0 underwent, at least two months after biopsy, a multiparametric contrast enhanced 3 T MRI. Patients were selected if the positive biopsy pattern was in complete concordance with the PCa lesions identified by MRI with precise loci matching on multimodal approach. Exclusion criteria included clinically bilateral cancer, biopsy-proven extraprostatic extension of cancer, evidence of metastatic or nodal disease on bone scan or cross-sectional imaging, prior significant rectal surgery preventing insertion of transrectal HIFU probe, any contraindication for pelvic MRI or anaesthesia, presence of prostatic calcification and cysts whose location will interfere with effective delivery of HIFU therapy, biopsy/MRI discordance, and latex allergies as the HIFU probe is covered with a latex condom sheath.

Any short term pretreatment androgen deprivation therapy (ADT) and/or 5*α*-reductase inhibitors (5-ARI) that had been given by referring physicians at the outside institution was discontinued at study entry. All patients gave preoperative consent after detailed discussion of limitations and benefits of hemiablation HIFU for the known clinically unilateral PCa and the need for long term followup with the intention to treat recurrent or progression lesion or de novo contralateral cancer.

Hemiablation HIFU was defined as ablation of one lobe of the prostate and not just the index lesion because of device technical limitations. It is, therefore, a region targeted therapy with emphasis on preserving controlateral neurovascular bundle, bladder neck, and external sphincter regardless of individual lesion grade, volume, or location and proximity to an ipsilateral neurovascular bundle.

All patients underwent hemiablation using HIFU delivered by the Ablatherm system (EDAP-TMS, Vaulx-en-Velin, France), performed by a single surgeon with a high level of experience in whole-gland HIFU. The procedure was done predominantly under spinal anaesthesia with a small proportion undergoing general anaesthesia. An 18 Fr Foley catheter was inserted prior to the procedure to drain the bladder and the catheter was clamped during the procedure. The boundaries of the prostate lobe to be treated were identified and accurately defined in the sagittal and transverse planes using integrated ultrasound imaging. A safety margin of 6 mm was maintained between the anatomical apex and the lowest section of the treated lobe; a safety margin of 4 mm was used when biopsies were positive at the apex. On the medial side the last zone to be ablated was mostly placed at the urethra identified by visualisation of the Foley catheter on the ultrasound image. On the lateral aspect the margin was set at the capsule of the prostate. The treatment progressed in multiple blocks depending upon the volume of the treated lobe. At the end of the procedure, a limited transurethral resection of the treated lobe was performed to prevent early acute urinary retention as well as sloughing of necrotic material requiring prolonged need for indwelling catheter. The Foley catheter was removed on the second postoperative day and the patient was discharged after establishing satisfactory voiding pattern.

Complications were prospectively recorded and graded according to the Clavien-Dindo score [14, 15]. Postoperatively, patients were followed with serial serum PSA determinations and digital rectal examinations at 1, 3, 6, and 12 months and then every 6 months. Given the presence of an untreated half prostate, an individual PSA nadir was identified in each patient. Followup also included whole-gland biopsies performed in the event of a PSA rising >2.0 ng/mL above nadir value (Phoenix criteria) [16]. Treatment failure was defined as positive biopsy of the treated area or if salvage or hormonal therapy was needed during followup. Urinary functional outcomes were reported using physician reported rates Overall QOL and costs were not reported in this study.

Kaplan-Meier analysis was performed to determine biochemical survival with failure defined according to Phoenix criteria. Local control and morbidity data are presented with descriptive statistics.

## 3. Results

**Figure 1:**
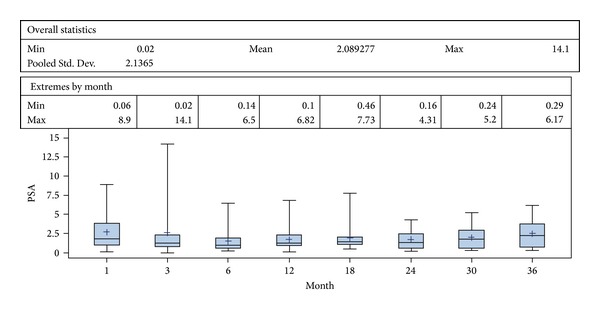
PSA dynamics during followup after hemi-HIFU treatment.

**Figure 2:**
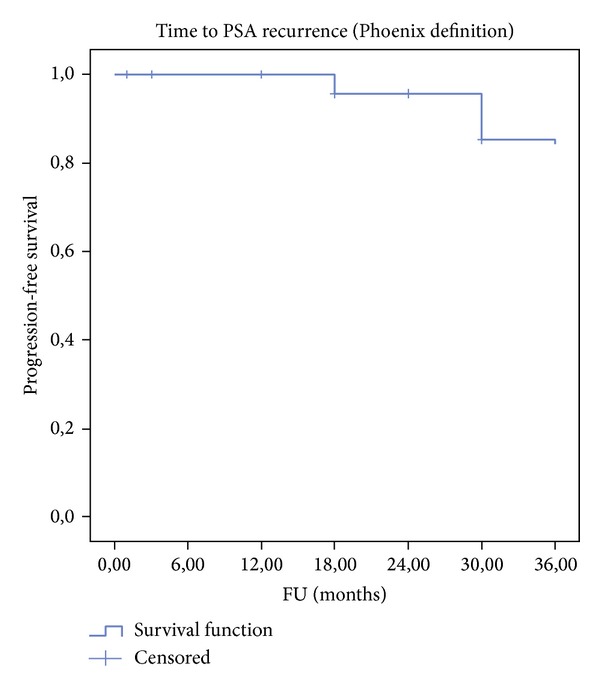
Biochemical progression-free survival (Phoenix definition-Nadir + 2 ng/mL).

Baseline characteristics of the study population are summarized in Table 1. Overall, a total of 31 patients (average age 71 years) were enrolled in this study with a mean followup of 36.3 months and a median followup of 38 months (range 12–61 months). The mean value of presenting PSA was 5, 67 ng/mL (range 0.3–11 ng/mL). Seven patients had a clinical stage T1c and the rest had cT2a or cT2b. Gleason score ranges from 5 to 9. With respect to the loci of cancerous lesion, 12 patients had apical disease, 17 had basal lesion, and 2 patients had exclusive transition zone lesions. Patients were stratified to risk groups according to D'Amico classification [17]. The median pre- and posttreatment prostate volume was 28.8 cc and 16.4 cc, respectively. The median length of hospital stay was 4 days due to local reimbursement practice and preoperative evaluation. The perioperative data are summarized in Table 2. The incidences of the most frequent complications; namely, acute urinary retention, urinary tract infection, lower urinary tract symptoms (LUTS), and urethral stricture, were reported in Table 3. Regarding grade 1 and grade 2 complications, six patients (19.3%) had self-resolving hematuria and LUTS, three patients (9.7%) had urinary tract infection, one patient (3.2%) had acute urinary retention, and one patient had voiding LUTS treated by anticholinergics (3.2%). A grade 3b complication occurred in one man (3.2%) who had a urethral stricture managed by endoscopic urethrotomy. No patient presented any grade 4 or higher complication. Urinary functional outcomes were reported using physicians reported rates. Two patients reported transient stress urinary incontinence during their first-month posttreatment visit. This resolved at the 3-month visit spontaneously and all patients were continent at their last followup giving a long term pad free continence rate of 100%. Two patients needed early hormone therapy due to PSA failure and data were lacking in nine patients for pre- and postoperative erectile function. In preoperatively potent patients (*n* = 20), 4 men (25%) had documented posthemiablation erectile dysfunction (ED) and 16 men (75%) had erections satisfactory for sexual intercourse. The mean age of patients with postoperative ED was 71.5 years (69, 71, 71, and 76 years). The median time to return of normal erection was 4.5 months (range 1–12 months) in patients with satisfactory sexual intercourse posttreatment. If we consider the nine patients with lacking data to have ED posttreatment, the ED rate for this cohort posttreatment would be 44.8% (13/29) and 55.2% (16/29) had erectile function sufficient for penetration. No patient developed metastasis or lymph node disease or died during followup. The mean (median) [range] PSA nadir was 1.49 ± 2.0 (0.93) [0–8.9] ng/mL and the mean (median) [range] time to achieve PSA nadir was 15.3 ± 9.6 (13.0) [4–39] weeks. The mean PSA nadir posttreatment represents a fall of 74% from the initial value (iPSA). Out of the 31 patients enrolled, two understaged patients with a Gleason 9 showed rapid PSA rising above iPSA value posttreatment and were deemed as treatment failures and started on hormonal therapy immediately. In spite of a good initial response to HIFU and a PSA nadir observation, two patients followed at distant centres were lost for evaluation. Overall, 27/31 patients were suitable for midterm oncologic observations. During followup, 5/27 (18.5%) patients exhibited PSA elevation ≥2.00 ng/mL above nadir; they underwent a new set of bilateral biopsies, accordingly. Two patients showed a negative biopsy and 3 patients (11.1%) had positive contralateral biopsies warranting contralateral HIFU therapy; further followup of these 3 patients showed complete clinical response with new PSA nadir subsequently. The distribution of PSA for the whole study follow up was reported using the box-and-whiskers plots which indicate the median of PSA at the protocol defined dates of follow up with interquartile (boxes) and range (whiskers) (Figure 1). Overall, the mean biochemical recurrence-free survival was 100%, 89%, and 82.7% at 1, 2, and 3 years, respectively, with overall and cancer specific survival of 100% at median follow up of 38 months (Table 4 and Figure 2).

## 4. Discussion

Many case series have reported encouraging short term functional and oncological results of men with PCa treated primarily in a focal manner [18–28]. To our knowledge, our study is the first midterm report (median follow up of 38 months) of the focal therapy on a cohort of patients primarily treated by hemiablation HIFU for a clinically unilateral PCa. Hemiablation HIFU of an entire lobe delivered with the intention to treat is feasible and functional and disease control outcomes are encouraging at 3 years of follow up. The principle rationale of tissue preservation is harm reduction. Early self-resolving LUTS were the most common complications. In addition, no rectal toxicities were reported and the strategy was well tolerated in the genitourinary functional domains. The procedure could possibly be delivered in an ambulatory care setting; the long stay of 4 days in our series is related to local reimbursement practice, preoperative anaesthetic evaluation, and transurethral partial resection of the prostate. Tissue preservation leads to functional preservation: all patients were pad-free continent despite a high number of apical lesions (*n* = 12) and only 25% of men in our cohort of relatively elderly patients (average age 71 years) who were potent preoperatively reported having ED after hemiablation. This treatment strategy is associated with a good medium term cancer control in well-selected patients (unilateral low to intermediate risk PCa). In the presented trial, assessment of oncologic efficacy was performed by serial PSA testing and random systematic TRUS guided biopsies were offered only for a cause (Phoenix criteria) in order to minimise burden on the patient. Furthermore, performance of systematic biopsies in treated and untreated lobes in all patients may increase the cancer detection rates during follow up but the clinical implication of such a protocol is unknown because it may simply reveal small foci of low grade low volume PCa. In the literature, there is no consensus on whether cancer control in focal therapy should be considered the absence of any cancer or the absence of clinically significant cancer and whether this should be limited to the treated or untreated area. In addition there is no standard follow-up protocol for the assessment of clinical failure. In our opinion, histologic confirmation of complete ablation within the treated area appears to be essential in focal therapy. That is why any positive biopsy in the treated lobe independently of the percentage of core involvement was considered a clinical failure. Controlateral positive biopsy was not considered as a clinical failure but as a technical limitation and was treated by a secondary controlateral hemiablation according to our protocol. A shift to systemic or salvage procedure was considered as a treatment failure. Phoenix criteria were not considered as response criteria, in our series, to define failure but as a threshold to offer biopsy. In our opinion, the only valid endpoint with a follow-up >1 year is the PSA nadir and the biopsy should be offered for a defined reason (BCR or a suspicious lesion on multiparametric MRI). As a surrogate, although PSA testing is accepted as a valid outcome in standard therapies, the clinical utility of PSA kinetics in tissue preservation is yet to be determined because many factors influence posttreatment values (the proportion of initial PSA that was due to tumor, amount of residual prostate tissue, progression of BPH, and TURP…). In our series, there was a 74% decrease in PSA levels from baseline which indicates successful ablation of the index lesion based on prior data that the index cancer accounts for 80% of entire cancer volume in a given patient [29]. The time to achieve PSA nadir was between 3 and 6 months. Biochemical recurrence- (BCR-) free survival was 100%, 89%, and 82.7% at 1, 2, and 3 years, respectively, which is comparable to standard therapies. In low to intermediate risk PCa, we noted no progression to metastatic or lymph node disease with overall and cancer specific survival of 100% at median follow up of 38 months. No residual tumour was noted in the treated area when biopsy was performed which gives us a recurrence rate of 0%. Three patients had positive contralateral biopsies treated by a secondary contralateral hemiablation with complete clinical response, a new PSA nadir, and without complications. Secondary treatment to the other lobe is an advantage of the technique and should not be considered as a failure because, at that point in time, any whole-gland therapy would be considered as a failure. Two patients needed systemic therapy and were considered as failures but these patients were at high risk of progression (Gleason 9) at initial setting. Accordingly, hemiablation HIFU should be indicated only for patients with localised PCa at low and intermediate risk of progression [30].

There are several limitations to our study. First, the safety and functional and oncologic outcomes are the results of a single centre with a long experience with whole-gland HIFU and cannot be generalized. The outcomes could be variable in less experienced hands because HIFU is a dynamic therapy with real time feedback which is difficult to master while assessing quality control. Even for whole-gland HIFU, results were significantly different between experienced and nonexperienced centres [31]. Second, the application of a limited TURP may have contributed to the toxicity seen above and beyond that of hemiablation HIFU. Third, the number of participants included in the study was small but as a prospective feasibility study we designed our trial to primarily assess feasibility. Fourth, our study was slow to recruit and heterogeneous with two Gleason 9 patients. Fifth, the use of disease specific and overall survival would require large scale randomized controlled trials with longer follow up to obtain sufficient evidence to prove noninferiority over radical whole-gland therapies or superiority over active surveillance. Sixth, the cohort was small with a short follow up and no control group to assess collateral damage and functional and oncologic outcomes. Apart from feasibility, this study provides level 4 evidence from which limited conclusions should be drawn.

## 5. Conclusions

The role of focal therapy in primary treatment of prostate cancer is best described as experimental and promising as progressively more and more studies are reporting good results. Our study suggests that hemiablation HIFU is a valid focal therapy strategy, feasible in day-to-day practice with good promising results. Well-designed, multicenter, prospective, randomized controlled studies are required to definitely establish the role of hemiablation and focal therapies as the standard of care in prostate cancer. The eventual success of these therapies, however, will depend not only on the form of focal therapy but also mainly on technological advances in imaging and diagnostic techniques improving diagnostic and tumour localization accuracy.

## Figures and Tables

**Table 1:** Baseline characteristics.

Mean age (median) [range]	70.9 ± 6.2 (71) [55–83]
Mean PSA ng/mL (median) [range]	5.67 ± 3.1 (5.3) [0.3–11.0]
Mean Gleason, (median) [range]	6.3 ± 1.0 (6.0) [4–9]
Hormone, *n* (%)	
Yes	3 (15.6)
No	28 (84.4)
Stage, *n* (%)	
T1	7 (22.5)
T2	24 (77.5)
D'Amico risk group*, *N* (%)	
Low	17 (54.8)
Intermediate	12 (38.7)
High	2 (6.5)
Gleason score, *n* (%)	
≤6	19 (61.3)
=7	10 (32.2)
≥8	2 (6.5)

*Risk group based on D'Amico definition (according to stage, Gleason, and PSA).

**Table 2:** Perioperative data.

Mean prostate volume, cc (median) [range]	28.4 ± 10.0 (28.4) [11.5–50.1]
Mean treated volume, cc (median) [range]	16.2 ± 5.3 (16.2) [6.0–28.3]
Mean treated ratio, % (median) [range]	58.4 ± 10.2 (58.0) [39.2–75.1]
Mean number of lesions, (median) [range]	301 ± 88 (289) [182–559]
Mean hospitalization duration, days (median) [range]	4 ± 0.8 (4) [2–6]
Mean catheterization duration, days (median) [range]	2.8 ± 3.9 (2) [2–21]

**Table 3:** Adverse events.

Adverse events (number of patients)	Management
Urinary tract infection (3)	
Prostatitis (2)	ATB (IV for 48 h than per os)
Balanitis (1)	ATB (Topical)
Acute urinary retention (1)	
Tissue sloughing	Indwelling urinary catheter one month
Lower urinary tract symptoms (5)	
Voiding LUTS (2)	Spontaneous resolution
Storage LUTS (3)	1 patient long term anticholinergics
Hematuria (5)	Oral hydration
Urethral stricture (1)	Optical urethrotomy
Incontinence (2)	
Urge incontinence	Spontaneous resolution
Stress urinary incontinence	Spontaneous resolution
Erectile dysfunction (4)	PDE5I* or intracavernous injection

*PDE5I: Phosphodiesterase type 5 inhibitors.

**Table 4:** Actuarial biochemical results.

Biochemical progression free survival rate*, % (total number at risk = 29)	
1-year	100
2-year	89
3-year	82.7

*Phoenix definition (nadir +2 ng/mL).

## References

[1] Jemal A, Siegel R, Ward E, Hao Y, Xu J, Thun MJ (2009). Cancer statistics, 2009. *CA: A Cancer Journal for Clinicians*.

[2] Marberger M, Barentsz J, Emberton M (2012). Novel approaches to improve prostate cancer diagnosis and management in early-stage disease. *BJU International*.

[3] Kazer MW, Psutka SP, Latini DM, Bailey DE (2013). Psychosocial aspects of active surveillance. *Current Opinion in Urology*.

[4] Wilt TJ, Brawer MK, Jones KM (2012). Radical prostatectomy versus observation for localized prostate cancer. *The New England Journal of Medicine*.

[5] Boorjian SA, Eastham JA, Graefen M (2012). A critical analysis of the long-term impact of radical prostatectomy on cancer control and function outcomes. *European Urology*.

[6] Resnick MJ, Koyama T, Fan KH (2013). Long-term functional outcomes after treatment for localized prostate cancer. *The New England Journal of Medicine*.

[7] Turpen R, Rosser CJ (2009). Focal therapy for prostate cancer: revolution or evolution?. *BMC Urology*.

[8] Valerio M, Ahmed HU, Emberton M (2013). The role of focal therapy in the management of localised prostate cancer: a systematic review. *European Urology*.

[9] Beerlage HP, Van Leenders GJLH, Oosterhof GON (1999). High-intensity focused ultrasound (HIFU) followed after one to two weeks by radical retropubic prostatectomy: results of a prospective study. *Prostate*.

[10] Dickinson L, Hu Y, Ahmed HU (2013). Image-directed, tissue-preserving focal therapy of prostate cancer: a feasibility study of a novel deformable magnetic resonance-ultrasound (MR-US) registration system. *BJU International*.

[11] Biermann K, Montironi R, Lopez-Beltran A, Zhang S, Cheng L (2010). Histopathological findings after treatment of prostate cancer using high-intensity focused ultrasound (HIFU). *Prostate*.

[12] Ryan P, Finelli A, Lawrentschuk N (2012). Prostatic needle biopsies following primary high intensity focused ultrasound (HIFU) therapy for prostatic adenocarcinoma: histopathological features in tumour and non-tumour tissue. *Journal of Clinical Pathology*.

[13] Gore JL, Shariat SF, Miles BJ (2001). Optimal combinations of systematic sextant and laterally directed biopsies for the detection of prostate cancer. *Journal of Urology*.

[14] Dindo D, Demartines N, Clavien PA (2004). Classification of surgical complications: a new proposal with evaluation in a cohort of 6336 patients and results of a survey. *Annals of Surgery*.

[15] Mitropoulos D, Artibani W, Graefen M, Remzi M, Rouprêt M, Truss M (2012). Reporting and grading of complications after urologic surgical procedures: an ad hoc EAU guidelines panel assessment and recommendations. *European Urology*.

[16] Roach M, Hanks G, Thames H (2006). Defining biochemical failure following radiotherapy with or without hormonal therapy in men with clinically localized prostate cancer: recommendations of the RTOG-ASTRO Phoenix Consensus Conference. *International Journal of Radiation Oncology Biology Physics*.

[17] D’Amico AV, Whittington R, Bruce Malkowicz S (1998). Biochemical outcome after radical prostatectomy, external beam radiation therapy, or interstitial radiation therapy for clinically localized prostate cancer. *Journal of the American Medical Association*.

[18] Onik G, Vaughan D, Lotenfoe R, Dineen M, Brady J (2008). The “male lumpectomy”: focal therapy for prostate cancer using cryoablation results in 48 patients with at least 2-year follow-up. *Urologic Oncology*.

[19] Lambert EH, Bolte K, Masson P, Katz AE (2007). Focal cryosurgery: encouraging health outcomes for unifocal prostate cancer. *Urology*.

[20] Muto S, Yoshii T, Saito K, Kamiyama Y, Ide H, Horie S (2008). Focal therapy with high-intensity-focused ultrasound in the treatment of localized prostate cancer. *Japanese Journal of Clinical Oncology*.

[21] Ward JF, Jones JS (2012). Focal cryotherapy for localized prostate cancer: a report from the national Cryo On-Line Database (COLD) Registry. *BJU International*.

[22] El Fegoun AB, Barret E, Prapotnich D (2011). Focal therapy with high-intensity focused ultrasound for prostate cancer in the elderly. A feasibility study with 10 years follow-up. * International Brazilian Journal of Urology*.

[23] Ahmed HU, Hindley RG, Dickinson L (2012). Focal therapy for localised unifocal and multifocal prostate cancer: a prospective development study. *The Lancet Oncology*.

[24] Barret E, Ahallal Y, Sanchez-Salas R (2013). Morbidity of focal therapy in the treatment of localized prostate cancer. *European Urology*.

[25] Ahmed HU, Freeman A, Kirkham A (2011). Focal therapy for localized prostate cancer: a phase I/II trial. *Journal of Urology*.

[26] Bahn DK, Silverman P, Lee F, Badalament R, Bahn ED, Rewcastle JC (2006). Focal prostate cryoablation: initial results show cancer control and potency preservation. *Journal of Endourology*.

[27] Ellis DS, Manny TB, Rewcastle JC (2007). Rewcastle cryoablation as primary treatment for localized prostate cancer followed by penile rehabilitation. *Urology*.

[28] Bahn D, de Castro Abreu AL, Gill IS (2012). Focal cryotherapy for clinically unilateral, low-intermediate risk prostate cancer in 73 men with a median follow-up of 3.7 years. *European Urology*.

[29] Ohori M, Eastham JA, Koh H, Kuroiwa K, Slawin K, Wheeler T (2006). Is focal therapy reasonable in patients with early stage prostate cancer (CAP)? An analysis of radical prostatectomy (RP) specimens. *The Journal of Urology*.

[30] Taneja SS, Mason M (2010). Candidate selection for prostate cancer focal therapy. *Journal of Endourology*.

[31] Baumunk D, Andersen C, Heile U (2013). High-intensity focussed ultrasound in low-risk prostate cancer—oncological outcome and postinterventional quality of life of an inexperienced therapy centre in comparison with an experienced therapy centre. *Aktuelle Urologie*.

